# Unraveling the Immune Microenvironment in Diffuse Large B-Cell Lymphoma: Prognostic and Potential Therapeutic Implications

**DOI:** 10.3390/cimb46070420

**Published:** 2024-07-05

**Authors:** Epameinondas Koumpis, Alexandra Papoudou-Bai, Konstantina Papathanasiou, Evangelos Kolettas, Panagiotis Kanavaros, Eleftheria Hatzimichael

**Affiliations:** 1 Department of Hematology, Faculty of Medicine, School of Health Sciences, University of Ioannina, 45 500 Ioannina, Greece; an.koumpis@uoi.gr (E.K.); konppth@gmail.com (K.P.); 2 Department of Pathology, Faculty of Medicine, School of Health Sciences, University of Ioannina, 45 500 Ioannina, Greece; apapoudou@uoi.gr; 3 Laboratory of Biology, Faculty of Medicine, School of Health Sciences, University of Ioannina, 45 110 Ioannina, Greece; ekoletas@uoi.gr; 4 Biomedical Research Institute, Foundation for Research and Technology, 45 110 Ioannina, Greece; 5 Department of Anatomy-Histology-Embryology, Faculty of Medicine, School of Health Sciences, University of Ioannina, 45 110 Ioannina, Greece; pkanavar@uoi.gr

**Keywords:** DLBCL, microenvironment, TME, lymphoma

## Abstract

Diffuse large B cell lymphoma (DLBCL) is a multifaceted condition characterized by significant diversity in its molecular and pathological subtypes and clinical manifestation. Despite the progress made in the treatment of DLBCL through the development of novel drugs, an estimated one-third of patients encounter relapse or acquire refractory disease. The tumor microenvironment (TME) of DLBCL, a complex network consisting of cellular and noncellular components that engage in interactions with the tumor, is a parameter that is gaining increasing attention. The TME comprises both the immune and nonimmune microenvironments. The immune microenvironment comprises natural killer (NK) cells, dendritic cells (DCs), tumor-associated macrophages (TAMs), neutrophils, myeloid-derived suppressor cells (MDSCs), and T and B lymphocytes. The nonimmune microenvironment consists of the extracellular matrix (ECM), cancer-associated fibroblasts (CAFs), mesenchymal stromal cells, and other molecules that are secreted. Despite ongoing research, the exact impact of these components and their interaction on the progression of the disease remains elusive. A comprehensive review of significant discoveries concerning the cellular and noncellular constituents, molecular characteristics, and treatment response and prognosis of the TME in DLBCL, as well as the potential targeting of the TME with novel therapeutic approaches, is provided in this article.

## 1. Introduction

Diffuse large B cell lymphoma (DLBCL), which accounts for almost 30% of all non-Hodgkin lymphomas, is a heterogeneous and aggressive disease showing differences in clinical presentation, pathological characteristics and molecular features [1,2,3,4,5,6,7]. Despite the addition of polatuzumab vedotin, an antibody–drug conjugate targeting CD79b, in the frontline treatment, still about one out of three patients experience relapse or refractory disease (R/R) [2].

DLBCL can be categorized as germinal center B-cell-like (GCB), activated B-cell–like (ABC), and unclassifiable DLBCL, based on the cell of origin, representing different B-cell development stages. The last two groups, ABC and unclassifiable DLBCL, are often combined and referred to as non-GCB [3,4]. GCB DLBCL is derived from normal germinal center B cells and is characterized by the expression of CD10, a cell membrane zinc-dependent metalloendopeptidase, and BCL6, a transcription factor regulating T follicular helper cells (TFH cells) proliferation with clinical significance in lymphomas, and also by *BCL2* gene rearrangements. ABC DLBCL is derived from peripheral activated B cells, being characterized by chronic B-cell receptor signaling, the activation of nuclear factor-kappaB (NF-κB), and IRF4/MUM1 expression [3,4]. This categorization, according to the B-cell differentiation stages, also has prognostic value, since patients with GCB-DLBCL display better overall survival (OS) compared to ABC patients [3]. In routine histopathology, immunohistochemistry-based algorithms, such as the Hans algorithm, are used to classify cases as GCB or non-GCB, with a risk of misclassification [5]. Interestingly, immunohistochemical studies using GC and non-GC B-cell differentiation immunophenotypes are useful not only for pathological classification, but also for further understanding of the pathogenesis of DLBCL. Indeed, (a) DLBCL with a GCB-cell-like immunophenotype has been significantly correlated with increased apoptosis, high expression levels of the pro-apoptotic proteins Bax, Bak, and Bid, and low expression levels of the anti-apoptotic protein Bcl-xl; and (b) DLBCL with increased expression levels of the GC-associated markers i.e., BCL6 and CD10 proteins, has been significantly correlated with increased apoptosis and proliferation of tumor cells [8,9]. Moreover, high expression of phosphorylated-c-Jun (p-c-Jun), JunB, JunD has also been positively correlated with the proliferation of DLBCL tumor cells [10]. More recent studies have proposed new molecular taxonomies for DLBCL, based on shared genomic aberrations [6,7]. Based on a multiplatform analysis of structural genomic abnormalities and gene expression in 574 DLBCL biopsy samples, a molecular classification of DLBCL was proposed that includes four subtypes, termed the MCD (*MYD88L265P-* and *CD79B*-co-mutated) subtype, the BN2 (*BCL6*-fusions or *NOTCH2*- mutated) subtype, the N1 (*NOTCH1*-mutated) subtype, and the *EZH2* (based on *EZH2* mutations and *BCL2* translocations) subtype, whereas a significant number of cases remained unclassified [7]. The MCD and N1 subtypes are dominated by ABC cases, the EZB subtype includes mostly GCB cases, and the BN2 subtype has contributions from all three gene-expression subgroups [7]. Another genomic analysis of 304 cases of DLBCL identified five different molecular signatures or clusters (C) [6]. In particular, the C1 signature was associated with *NOTCH2* mutations and a favorable outcome; C2 was associated with aneuploidy; *TP53* was associated with biallelic inactivation and a poor outcome; C3 was characterized by *BCL2* mutations, translocations, and mutations in the chromatin modifiers and was associated with an unfavorable outcome; C4 was associated with abnormalities affecting signaling pathways, such as RAS/JAK/STAT, and a favorable outcome; and C5, which includes cases with 18q gains and *MYD88* and *CD79B* mutations, was associated with a poor outcome [6]. A distinct cluster, termed C0 by the investigators, lacked defining genetic drivers and included increased numbers of cases of T-cell/histocyte-rich large B-cell lymphomas indicating the different pathobiology of this condition (Table 1) [6].

**Figure 1:**
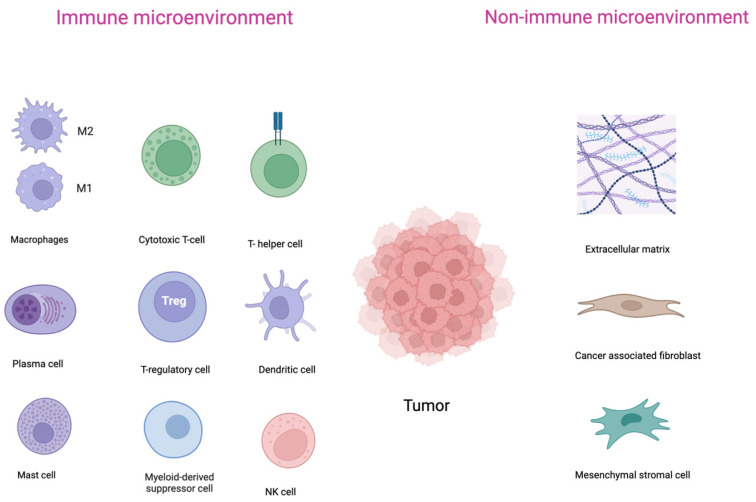
Components of immune and nonimmune microenvironment in DLBCL.

Another crucial factor in the pathobiology of DLBCL, with a possible prognostic and predictive values, is the tumor microenvironment (TME) [11]. The TME is a complex biological network that consists of interacting cellular and noncellular components, surrounding the tumor and interacting with it, and therefore plays a crucial role in tumor genesis, maintenance, and progression [11,12]. The TME consists of the immune microenvironment, which includes immune cells such as T and B lymphocytes, natural killer (NK) cells, tumor-associated macrophages (TAMs), neutrophils, myeloid-derived suppressor cells (MDSCs), dendritic cells (DCs); and the nonimmune microenvironment, composed of cancer-associated fibroblasts (CAFs), extracellular matrix (ECM), mesenchymal stromal cells, and other secreted molecules, such as chemokines and cytokines (Figure 1) [11,13].

The TME of DLBCL encompasses a variety of cell types, each comprising heterogeneous subsets with distinct phenotypes and functions [14,15]. The proportions of cell types and their functional state can be identified by flow cytometry and transcriptional signatures [14,16]. Artificial intelligence-based computational methods have delineated distinct “lymphoma microenvironments” (LMEs) in DLBCL, each exhibiting unique clinical and biological characteristics [14,15]. Specifically, the germinal center–like LME (GC-LME), which is characterized by the presence of cell subtypes commonly found in germinal centers; the mesenchymal LME (MS-LME), which is dominated by stromal cells and extracellular matrix signatures; the inflamed and immunosuppressive LME (IN-LME), which is characterized by the presence of inflammatory cells and suppressed cytotoxic cells; and the depleted LME (DP-LME), which has less prominent TME-derived signatures [14,15].

Regarding the therapeutic strategy in newly diagnosed DLBCL, either rituximab in combination with cyclophosphamide, doxorubicin hydrochloride, vincristine sulfate, and prednisone (R-CHOP) or polatuzumab vedotin in combination with rituximab, cyclophosphamide, doxorubicin hydrochloride, and prednisone (Pola-RCHP) are used [2,17]. For the primary refractory and early relapsed DLBCL (<1 year of the treatment completion) chimeric antigen receptor (CAR) T-cell therapy is the recommended treatment, whereas for late relapses, either salvage chemotherapy followed by autologous transplantation or CAR T-cell therapy is recommended [17]. Other treatment options for patients that are not eligible for transplant include the combination of polatuzumab vedotin with rituximab and bendamustine, tafasitamab with lenalidomide, and, upon second relapse, bispecific antibodies, namely glofitamab and epcoritamab [17,18].

In this review, we summarize important research findings regarding the cellular and molecular composition of the TME in DLBCL, and we scrutinize its role in the prognosis, treatment response, and potential targeting.

## 2. Overview of Cellular Components of TME in DLBCL

### 2.1. T Cells

The T cells in the TME of lymphomas have been extensively studied due to their abundance and versatile functions (Figure 1). Effective B cell-mediated immunity and antibody responses often rely on the contribution of CD4+ T cells, particularly T follicular helper (TFH) cells, which are regulated by the transcription factor BCL6 [19]. TFH cells are identified by the expression of cell surface markers CD4, CXCR5, PD1, and ICOS. They primarily reside in the lymph nodes, tonsils, and spleen, playing a crucial role in germinal center formation and maintenance by providing critical helper signals, such as CD40L [20,21,22]. In addition, TFH cells promote the clonal selection and affinity maturation of GCB cells [20,21]. The expression of the chemokine receptor CXCR5 is linked to the early TFH cell migration to the border of the B cell follicle, whereas Th1, Th2, or Th17 signals lead to Th1, Th2, or Th17 CD4 cell differentiation programs, driving the effector cell outside the lymphoid tissue [20].

CD4+CXCR5+FOXP3+ follicular regulatory T (TFR) cells play a crucial role in downregulating the germinal center reaction, B cell activation, and TFH cytokine secretion [19]. TFR cells were found to be functionally different from non-TFR T regulatory (Treg) cells. Patients with less advanced DLBCL stages and those who stayed in remission 2 years after the initial chemoimmunotherapy treatment showed higher amounts of TFR cells within the tumor [23]. Lymphoma-infiltrating TFH cells were associated with high levels of certain cytokines, such as IL-4, IL-6, IL-21, and CXCL13 [21]. Significantly increased TFH cell ratios were observed in patients with malignant lymphoma disease at pretreatment compared to healthy controls, and decreased, or even normal, TFH cells ratios were observed in patients at the end of treatment [24]. However, in cases of progressive disease, elevated levels of TFH cells were noticed, indicating their crucial role [24].

FOXP3+Treg cells play a role in maintaining immunological tolerance and homeostasis, and they are also implicated in the TME of DLBCL. These cells limit T-helper-cell (TH)-mediated immune responses by releasing immunosuppressive cytokines, which help maintain self-tolerance while hindering anti-tumor immunity [25].

Importantly, due to limitations in identifying this specific cellular population, we should interpret results on the prognostic significance of Treg cells with caution [26]. FOXP3 is considered the best marker for Treg cells [27,28]. However, human non-regulatory CD4+ or CD8+ T cells have the ability to express FOXP3 [29,30] and, upon activation, a majority of human FOXP3−CD25− T cells can temporarily acquire the characteristics of Treg cells, such as the co-expression of FOXP3 and CD25, as well as the ability to suppress the proliferation of autologous CD4+CD25− T cells [31]. Two retrospective studies have found that a greater presence of intratumoral Treg cells is linked to prolonged OS and other positive prognostic factors, such as the absence of spleen enlargement and early-stage illness, in patients with DLBCL [32,33]. In a meta-analysis of fourteen studies, FOXP3+Treg cell expression was not associated with OS [34]. However, in the subgroup analysis, the authors found that higher expression of FOXP3+Treg cells was significantly correlated with better OS when the expression was measured by the number or percentage of positive cells instead of the score [34]. On the other hand, high levels of T cell immunoglobulin and mucin-containing molecule 3 (TIM-3)+FOXP3+Treg in the lymphoma microenvironment were associated with poor survival of DLBCL patients [35]. TIM-3+FOXP3+Treg cells could contribute to DLBCL development by secreting IL-10 in the TME, whereas antiTIM3 antibodies could be a potential future treatment regimen that blocks the secretion of IL-10 [35].

CD8+ T cells are generally known as cytolytic T cells (CTLs) due to their capacity to directly kill infected or neoplastic cells after recognizing antigens bound to MHC (major histocompatibility complex)-I molecules on their surface. Therefore, they are considered crucial mediators of anti-tumor immunity, alongside other major cytolytic cells, such as the NK cells [26]. Tumor-infiltrating lymphocytes (TILs) are essential members of the TME in DLBCL, and CD8+TILs are the main components that deliver anti-tumor immune response [36]. In cancer, tumor progression is induced by exhausted CD8+ T cells, a term that is used to describe T cells that undergo a progressive loss of cytokine production and cytotoxicity [37]. Low tumor-infiltrating T lymphocytes and a high CD4/CD8 ratio were associated with shorter survival in patients with DLBCL, indicating the crucial role of CD8+ T cells in the TME of DLBCL [38]. Despite the fact that PD-1 is expressed in TILs and was linked with shorter survival, the PD-1 blockade by nivolumab in patients with R/R DLBCL was ineffective [39,40]. Thus, the exploration of new immune checkpoints in DLBCL is important, as is the assessment of the potential efficacy of novel immune checkpoint inhibitors or combined blockade regimens [41]. New immune checkpoints in DLBCL include T cell immune receptors with Ig and ITIM domains (TIGIT), lymphocyte-activation-gene-3 (LAG-3), and TIM-3. TIGIT is a co-inhibitory receptor in the Ig superfamily, expressed by activated T cells, Treg, and NK cells. The persistent activation of cancer cells by antigens results in the ongoing production of TIGIT, leading to the depletion of T-cell activity [41]. LAG-3 is an immune inhibitory receptor, with MHC-II as a canonical ligand, and is mainly expressed in activated T cells, NK cells, and Treg cells [41,42]. Fibrinogen-like protein 1 (FGL-1) is a major LAG-3 functional ligand independent from MHC-II and induces a significantly reduced anti-tumor response [42]. Human cancer cells produce high levels of FGL1, and increased peripheral levels of FGL1 in cancer patients have been associated with a poor prognosis and resistance to anti-PD-1/B7-H1 therapy [42]. Elevated LAG-3 and PD-1 levels significantly inhibit CD8+ T-cell function, rendering them unable to kill tumor cells. Combined LAG-3 and PD-1 blockade could restore CD8+ T cell function and is a promising effective combination immunotherapy for DLBCL [43]. CD8+ T lymphocytes, CD4+ T lymphocytes, NK cells, and monocytes primarily express TIM3 [44]. In addition, overexpression of TIM3 has been associated with CD8+TIL exhaustion and immune deficiency in DLBCL [36,45]. It is suggested that Galectin-9 is the key ligand of TIM3-mediated CD8+TIL exhaustion in DLBCL [36].

### 2.2. B Cells and Plasma Cells

Among the infiltrating non-neoplastic cells of the TME in DLBCL, naïve B cells and plasma cells have been recognized, and their prognostic role has been investigated. A recent study of 269 people with DLBCL found that having more normal B cells (as shown by clonotype analysis) in the total B cells was linked to a much higher chance of survival in DLBCL [46]. A more recent study using flow cytometry in fresh biopsy tissues at the clinical presentation of 102 patients with DLBCL confirmed that the frequency of normal B cells in the TME of DLBCL was positively correlated with favorable clinical outcomes [16]. However, in another study of 539 samples with DLBCL, where investigators used CIBERSORT in the R software package, version 4.1.1, univariate Cox analysis showed that neither B cells nor plasma cells had any significant correlation with survival [47].

### 2.3. NK Cells

Natural killer (NK) cells are innate lymphoid cells with known anti-tumor cytotoxic activity. NK cells are also regulatory cells modulating interactions with dendritic cells, macrophages, T cells, and endothelial cells [48]. Moreover, NK cells can discriminate target cells such as cancer cells from healthy cells, expressing receptors that allow them to recognize pathogens and activate effector functions such as cytotoxicity and cytokine production [48,49]. Activating NK cell receptors find ligands on cells that are “in distress”, like the stress-induced self-ligands recognized by NKG2D. In addition, NK cells express certain Toll-like receptors (TLRs) [49]. In vitro, the exposure of NK cells to TLR ligands induces interferon (IFN)-γ production and enhances cytotoxicity, while in vivo, this process is more efficient when accessory cells are present [48,49]. The NK cell detection system also includes cell surface inhibitory receptors such as the MHC class I–specific receptors, especially killer cell immunoglobulin-like receptors (KIRs) and the lectin-like CD94-NKG2A heterodimers [50,51]. Dysregulation of NK cell function has a vital role in cancer development, since it leads to both the uncontrolled proliferation of cancer cells and the development of metastases [52,53]. NK cells, except for surface inhibitory receptors, express immune checkpoint molecules such as PD1, and overexpression of PD1 on NK cells has been detected in DLBCL [54]. The mechanism is more prominent in classical Hodgkin Lymphoma (cHL), compared to DLBCL; thus, PD-1 blocking is more efficacious in cHL and is employed as a treatment. Nevertheless, PD-1 inhibition may potentially serve as an advantageous intervention in DLBCL in the future [54].

### 2.4. Myeloid Cells

Myeloid-derived suppressor cells (MDSCs) represent a heterogeneous, immunosuppressive population of immature myeloid cells, and they play a crucial role in tumor progression [55,56]. They are divided into three distinct MDSC subcategories, including the monocytic, the granulocytic and non-monocytic, and the non-granulocytic MDSCs [57]. MDSCs are characterized by the expression of the myeloid markers CD11b and CD33 and low or absent expression of HLA DR, while the monocytic and granulocytic subsets of MDSCs are characterized by the expression of CD14 and CD15, respectively [58]. Generally, MDSCs are at low levels; however, they are expanded in conditions such as cancer and inflammation, and they are involved in lymphomagenesis [57,59,60,61]. In a recent retrospective study, elevated levels of M-MDSCs were observed in the peripheral blood of newly diagnosed and relapsed DLBCL patients, and in newly diagnosed patients, the frequency of M-MDSCs was positively correlated with tumor progression and negatively correlated with OS [55]. In addition, it was revealed that IL-35 mediated the accumulation of M-MDSCs in DLBCL patients, while anti-IL-35 treatment reduced the levels of M-MDSCs in mice, demonstrating their promising role as a potential therapy of DLBCL in the future [55].

Neutrophils are generally considered to be fully differentiated cells with specific functions and minimal plasticity; however, tumor-associated neutrophils (TANs), an important cellular component of the TME, are characterized by diversity and plasticity [62,63,64]. Neutrophils can have either pro-tumor or anti-tumor effects [63]. N1 neutrophils have anti-tumor properties, while N2 neutrophils exhibit pro-tumor characteristics. N1 neutrophils produce high levels of immune-activating cytokines and chemokines, demonstrating a stronger ability to kill tumor cells in vitro [65]. On the other hand, N2 neutrophils contribute to tumor progression within the TME by recruiting immunosuppressive CD4+ T cells and by upregulating CCL2, which enhances angiogenesis [65]. Circulating neutrophils enter tumors, where they differentiate into T1 and T2 TANs. T1 and T2 further differentiate to form the T3 subset. The T3 subset is characterized by dcTRAIL-R1 expression in mice, a significantly prolonged lifespan (more than 5 days), and proangiogenic and pro-tumoral functions [63,64]. Noteworthy, eliminating the factors responsible for the differentiation of T1/T2 into T3 does not reverse the T3 phenotype [64]. As far as DLBCL is concerned, immunohistochemical studies revealed that (a) 46% of DLBCL cases show upregulation of the proliferation-inducing TNF ligand (APRIL), which stimulates B-cell activation; and (b) neutrophils were the main source of APRIL in all the DLBCL cases with APRIL upregulation [66]. Moreover, malignant cells expressed the APRIL-signaling receptor, TACI and/or BCMA, indicating that these DLBCL cases are equipped to respond to APRIL [66]. A retrospective analysis of the clinical course revealed a statistically significant correlation between a high expression of APRIL in DLBCL lesions and a decreased overall survival rate of the patient [66]. Thus, APRIL produced by inflammatory cells, mainly neutrophils, infiltrating DLBCL lesions may increase the aggressiveness of the lymphoma and affect the outcome of the disease [66]. Furthermore, in a notable fraction of DLBCL patients, malignant cells constitutively produced the chemokine CXCL-8 (IL8), which enables them to recruit blood neutrophils that produce APRIL [67]. Thus, CXCL-8 derived from DLBCL cells can promote neutrophil infiltration, thereby providing a source of the tumor-promoting factor APRIL [67].

### 2.5. Mast Cells

Mast cells are a key regulatory component of the TME in DLBCL [68]. The infiltration of mast cells reflects the host inflammatory response, and elevated levels of mast cells have been associated with favorable clinical outcomes [69]. Moreover, in another study, the investigators observed that tryptase expression was significantly correlated with microvascular density, supporting a role for mast cells in DLBCL tumor angiogenesis [70].

### 2.6. Dendritic Cells

Dendritic cells (DCs) are another important cellular component of the TME in DLBCL. DCs are professional antigen-presenting cells capable of inducing naïve T-cell activation and effector differentiation [71]. In tissues, CD11c+ cells are mostly classical DCs [71]. Decreased CD11c+ dendritic cells in the DLBCL TME were an independent unfavorable prognostic factor, associated with shorter survival, as well as with the prediction of the presence of a double or triple hit genotype [72]. Increased proportions of DCs in the DLBCL TME were associated with favorable clinical outcomes [73]. Elevated levels of CD11c+ DCs in the peripheral blood of DLBCL patients have also been correlated with favorable OS [74].

### 2.7. Tumor-Associated Macrophages

**Figure 2:**
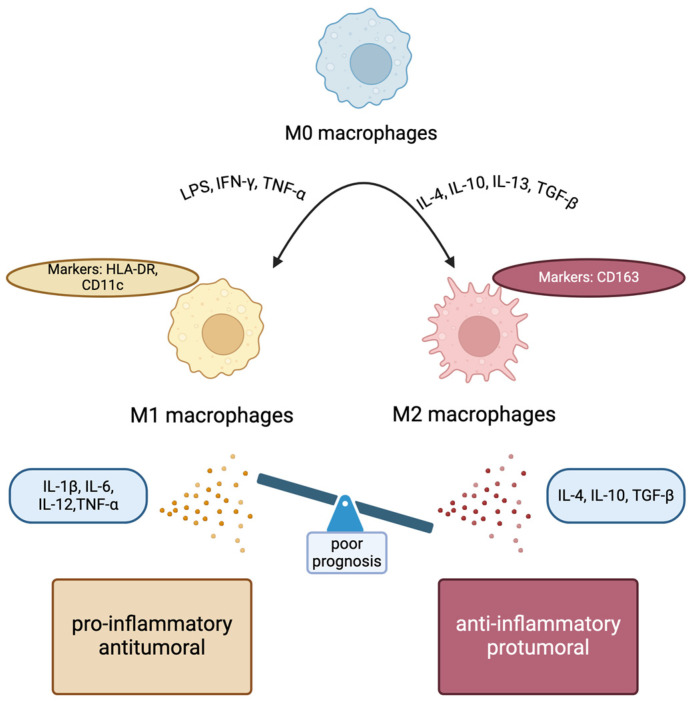
The polarization of macrophages in tumor microenvironment. LPS: lipopolysaccharides, IFN: interferon, TNF: tumor necrosis factor, IL: interleukin, TGF: tumor growth factor, HLA: human leukocyte antigen.

Tumor-associated macrophages (TAMs), located in the TME, are of great importance in contributing to cancer cell survival and progression [75]. TAMs can have either anti-tumorigenic (kill tumor cells) or pro-tumorigenic (promote tumor cell survival) effects [21]. They are classified into two distinct categories, based on their physical characteristics. Specifically, the M1 phenotype (CD68/HLA-DR), which is characterized by its anti-tumorigenic role via secreting proinflammatory cytokines (IL-1β, IL-6, IL-12, TNF-α, etc.), and the M2 phenotype (CD68/CD163), which is characterized by its pro-tumorigenic role via secreting anti-inflammatory cytokines (IL-10, IL-13, IL-4, matrix metalloproteinases, etc.) [21,76,77] (Figure 2).

TAMs are implicated in the pathogenesis of lymphomas, and they may have prognostic value in patients with DLBCL [78]. A study showed that an increased number of CD14+ monocytes with a loss of expression of human leukocyte antigen-DR (HLA-DR) in lymphoma patients was associated with more aggressive and R/R disease [79]. In another study, overexpression of CD68/CD163 TAMs (M2 phenotype) at diagnosis of DLBCL was associated with a poorer prognosis [80]. Nam et al. found that an increased ratio of CD163/CD68+ cells was an independent predictor of shorter OS and PFS in patients with DLBCL [81]. In another study, a higher number of M2 TAMs was an independent significant factor for poor prognosis [82]. However, some studies did not confirm the significant prognostic role of TAMs in DLBCL patients, as overexpression of CD68 TAMs did not significantly correlate to poorer clinical outcomes [83,84,85]. Another study highlighted the predictive value of TAMs. The investigators found that CD68+ TAM and CD68 mRNA levels were significantly correlated with a shorter OS in patients treated with CHOP; however, in patients treated with chemoimmunotherapy (Rituximab-CHOP), overexpression of CD68 was significantly correlated with a prolonged OS [86]. While most of the studies have focused on examining the potential prognostic role of macrophages in tissue biopsies of lymphoma patients, several very recent studies have highlighted their prognostic value as biomarkers, measured in the peripheral blood in patients with lymphoma [87,88,89]. A study showed that increased levels of serum soluble CD163 were associated with shorter OS in DLBCL patients [89]. Several clinical approaches targeting TAMs are still under investigation. Among them, the most promising target seems to be the blockade of CD47, which is overexpressed in lymphomas, including DLBCL [90,91]. CD47, or the integrin-associated protein, is a cell surface ligand normally expressed at low levels by nearly all cells of the body. Its role is crucial in DLBCL, where CD47 is overexpressed, providing a potent protection signal to macrophages, and thereby preventing phagocytosis [91,92]. Magrolimab, a humanized monoclonal antibody targeting the human cell surface antigen CD47, was an effective and tolerable treatment choice when it was combined with rituximab and chemotherapy in a phase 1b clinical study of patients with DLBCL. Further studies should be performed to confirm these data and explore the potential significance of incorporating magrolimab into the therapeutic schemes of DLBCL [93].

### 2.8. Cancer-Associated Fibroblasts

Resting fibroblasts are mesenchymal cells in the connective tissue [94]. They have been called “cockroaches of the human body”, since they can survive in severe, stressful conditions when all other cells cannot [89]. Resting fibroblasts can differentiate into active fibroblasts, which can generate growth factors and synthesize ECM [94]. Active fibroblasts are different from cancer-associated fibroblasts (CAFs), which contribute to tumorigenesis via enhanced migratory capacity, autocrine growth factor-induced signaling, and increased levels of secretory molecules [21,94]. CAFs are crucial modulators of tumor immunity, and they are a heterogeneous and plastic population within the TME [95]. Distinct CAF subtypes have been recognized, characterized by different molecular markers, such as myofibroblast-like CAFs (myCAFs), inflammatory CAFs (iCAFs), and antigen-presenting CAFs (ApCAFs), and they have distinct biological features and different roles in tumor development [95,96,97]. Subcategories of CAFs are not permanent, but interconvertible via manipulation of specific signaling, such as the conversion between iCAFs and myCAFs via the TGFβ- or IL-6 signaling pathway of CAFs [95]. Regarding DLBCL, the “stromal 1” gene signature shows enrichment in CAFs, with its expression inversely correlated to the tumor stage [21]. Therefore, CAFs theoretically contribute to the trapping of B cells in a specific anatomical location and preventing their spread [21,98]. The failure of clinical trials targeting CAFs highlights their plasticity and dynamic complexity, as well as the necessity of further studies to increase our understanding of CAF identity and function [95].

To sum up, the TME comprises various cellular constituents, including immune cells such as T cells, DCs, macrophages, and neutrophils, as well as cancer-associated fibroblasts. These components play crucial roles in tumor progression, immune evasion, and response to therapy, emphasizing their importance in comprehending and potentially targeting DLBCL.

## 3. Extracellular Matrix and Stromal Signature

The extracellular matrix (ECM) is a complex mixture of various proteins, mineral deposits, and proteoglycans produced by stromal cells. It serves to both support cells and regulate the interactions among them [78]. Genes that encode several ECM components, including collagen, laminin, and matricellular proteins, were linked to the “stromal-1 signature” and a better clinical outcome, as explained below [99].

More specifically, two gene expression signatures of non-malignant cells were described in patients with DLBCL [84]. These two subgroups were associated with different prognostic and predictive values, since the “stromal-1” response was correlated with a better prognosis, while an elevated expression of the “stromal-2” signature was associated with unfavorable outcomes and increased tumor blood vessel density [99]. Increased expression of the “stromal-1” signature was detected in tumors with abundant extracellular matrix elements and a high number of macrophages [99]. This signature encodes components of the ECM, including fibronectin, osteonectin, various collagen and laminin isoforms, and the antiangiogenic factor thrombospondin. In addition, it encodes modifiers of collagen synthesis (LOXL1 and SERPINH1), proteins that remodel the ECM (MMP2, MMP9, MMP14, PLAU, and TIMP2), and the connective-tissue growth factor (CTGF), a secreted protein that can initiate fibrotic responses [99,100]. In addition, the “stromal-1” signature comprises genes that are typically expressed in cells belonging to the monocytic lineage, such as the transcription factor *CEBPA* encoding the transcription factor C/EBPα (the CCAAT enhancer-binding protein alpha) and the *CSF2RA* encoding the colony stimulating factor 2 receptor subunit alpha [99]. Secreted protein acidic rich in cysteine (SPARC), also called osteonectin, is expressed by macrophages and plays an important role in the development of lymphoid malignancies, since it has been described as either a tumor suppressor or a tumor promoter [101,102]. In a cohort of 173 patients with DLBCL, the combined immunohistochemical assessment of fibronectin and SPARC was found to be an important tool for the prediction of survival [103]. Higher expression of each of them was associated with longer OS, and their combination had stronger prognostic significance [103]. The “stromal-2” signature includes markers of endothelial cells, such as von Willebrand factor (VWF) and CD31, or platelet endothelial cell adhesion molecule (PECAM-1), as well as other genes specifically expressed in endothelium, such as *EGFL7, MMRN2, GPR116,* and *SPARCL1*. Furthermore, this signature encodes key regulators of angiogenesis such as VEGF and genes expressed only in adipocytes, including *ADIPOQ, FABP4, RBP4,* and *PLIN* [99].

## 4. Immune Evasion of DLBCL

Various types of cancer employ immune evasion as a pathogenetic strategy during their progression. The primary mechanisms involved include the avoidance of circulating T lymphocytes or eluding detection by NK cells [78]. Multiple ways in which malignant cells manage to escape anti-tumor immune surveillance have been identified [104]. It is estimated that about three-quarters of DLBCLs carry genetic abnormalities in genes linked to immune evasion [6,7]. These gene aberrations associated with immune evasion are notably prevalent in the C1 and C5 subtypes, or in the MCD genetic subtype, are characterized by frequent occurrences of MYD88L265P and/or CD79B mutations, and are attributed to the ABC subtype [6,7]. The MHC-I expression plays a crucial role. MHC-I molecules are composed of a heavy (α) chain, encoded by HLA-I, and a light chain (β2-microglobulin, β2M), encoded by B2M [104]. Antigen-specific cytotoxic T lymphocytes engage with the MHC-I complex on target cells via a T-cell receptor (TCR) complex. Upon receiving a co-stimulatory signal, cytotoxic T cells become activated and proceed to eliminate the target cells [104,105]. The most prevalent mechanism associated with immune evasion is the absence of cell-surface expression of MHC-I, which is detected in approximately 50% of DLBCL cases [106]. In approximately 29% of DLBCL cases, genetic alterations can render the β2M gene inactive, thereby impeding the cell-surface presentation of the HLA-I complex, resulting in tumor cell evasion from cytotoxic T-cell surveillance [107]. Moreover, CD58, the receptor for NK cells and T cell CD2+, plays a crucial role in this evasion process. Inactivation of the *CD58 gene* was identified in 21% of DLBCLs, and it was notably more prevalent in the ABC subtype [107]. This inactivation leads to impaired recognition of tumor cells by cytotoxic T cells and NK cells [107]. The immune evasion process also includes reduced expression of the MHC-II, which presents tumor-specific antigens to CD4+ T cells [108]. Loss of MHC-II expression was correlated with decreased infiltration of T cells and a poorer prognosis [109].

The *CD70 *and* TNFSF9 genes*, which are suspected tumor suppressor genes, belong to the tumor necrosis factor superfamily [110]. CD70 engages with CD27 on T cells, initiating a signaling axis that promotes cell survival, boosts T-cell proliferation, and is believed to exert anti-tumor effects [111]. *TNFSF9* binds to *TNFRSF9* (CD137, 4-1BB) on activated T cells, thereby stimulating T-cell proliferation [112]. Aberrations on both these genes are detected in DLBCL, playing an important role in immune evasion [104]. As previously discussed, dysregulation of immune checkpoints, such as PD1, LAG3, and TIM-3, can lead to T-cell exhaustion. Novel immunotherapies (immune checkpoint inhibitors) are under investigation in patients with DLBCL [104,113]. Genetic disorders that impact genes responsible for modifying epigenetic processes are prevalent in DLBCL, with a detection rate of over 60% [6,7]. Such epigenetic modifier genes include the *histone-lysine N-methyltransferase 2D (KMT2D)*, the *enhancer of zeste homolog 2 (EZH2)*, the *cyclic-adenosine monophosphate response element-binding protein (CREBBP), *and* histone acetyltransferase p300 (EP300)* [104]. Genetic mutations that control epigenetic processes cause changes in the expression of several genes, affecting immune recognition molecules, response modifiers, and cytokines. The reorganization of the gene expression landscape is crucial in shaping the TME, leading to the progression of lymphoma. Several potentially effective treatment drugs that specifically target epigenetic modifiers are now being studied in patients with DLBCL [104,114]. Tazemetostat, a selective and orally available inhibitor of *EZH2*, has shown promising results in patients with R/R DLBCL in a phase 1 clinical study [115]. In another phase 1b study, tazemetostat was combined with atezolizumab, a monoclonal antibody targeting programmed death-ligand 1 (PD-L1) in patients with relapsed and refractory DLBCL. The combination was safe; however, the overall response rate was only 16% [116].

Histone deacetylases (HDACs) are a class of proteases that play an important role in the regulation of gene expression and are implicated in the development and drug resistance of lymphoma. When HDACs are abnormally expressed, they disrupt histone acetylation, resulting in the suppression of gene transcription and reduced CD20 expression, therefore mediating immune evasion [117,118]. HDAC inhibitors have shown promising results in DLBCL cell lines via the upregulation of CD20, enhancing the efficacy of anti-CD20 monoclonal antibodies and promoting lymphoma cell apoptosis [117]. Panobinostat, a pan-HDAC inhibitor, induced highly durable responses in certain patients with R/R DLBCL in a phase 2 clinical study [119]. Unfortunately, the results were not confirmed in another phase 2 clinical study, when panobinostat was tested as a single agent or in combination with everolimus in patients with R/R DLBCL [120]. Vorinostat, another HDAC inhibitor, has also been examined alone or in combination with pembrolizumab, showing promising results, mainly in patients with primary mediastinal DLBCL [121].

Interrelated intrinsic and extrinsic mechanisms may influence the highly complex interaction between malignant B cells and cellular components in the TME, ultimately leading to immune escape [21]. Cellular elements from both immune and stromal origins create complex cell-to-cell and paracrine networks with tumor B cells. This reciprocal modulation involves malignant clones and TAMs, as well as stromal and immune cells such as neutrophils, T cells, and DCs, through the expression of chemokines and cytokines and the deposition of ECM components [122]. In addition, accessory cells such as neutrophils and stromal cells can regulate tumor survival [66,123]. The cooperative interaction between neoplastic B cells and their supporting stromal cells facilitates and maintains cancer’s hallmarks, such as resistance to cell death (anti-apoptosis and drug resistance), sustained cell proliferation, angiogenesis, immune suppression, stemness and self-renewal, and cell homing and invasion, thereby promoting tumor progression [124].

## 5. Conclusions

Overall, the complex interaction between different immune cells in the TME of lymphomas, namely DLBCL, highlights the intricate nature of immune control and its impact on disease progression and patient outcomes. T cells, including CD4+ T cells and TFH cells, play pivotal roles in orchestrating immune responses, modulating B cell-mediated immunity, and determining the germinal center reaction. Treg cells, identified by FOXP3 expression, play a role in immunological tolerance and homeostasis, although their prognostic significance in DLBCL remains uncertain. Furthermore, immune checkpoint molecules such as PD-1 and TIM-3 mediate the exhaustion of CD8+ T cells, underscoring the necessity of investigating novel immune checkpoint inhibitors in DLBCL treatment strategies. In addition to T cells, other immune cell populations such as B cells, NK cells, MDSCs, mast cells, dendritic cells, and TAMs have substantial effects on tumor progression and patient outcomes. Notably, the ECM, the stromal signature, and the CAFs all play a part in how the TME is controlled and how it impacts the development of DLBCL, and how it responds to treatment. Recognizing the multifaceted interactions among immune cells, stromal components, and tumor cells offers exciting prospects for developing innovative therapeutic approaches targeting the immune landscape of DLBCL (Table 2).

## Figures and Tables

**Table 1:** Molecular subtypes of diffuse large B-cell lymphoma (LBCL), as defined by Chapuy et al.

Cluster (C)	Genetic Characteristics	Prognosis
C1	NOTCH 2 mutations	good
C2	aneuploidy and TP53 biallelic inactivation	poor
C3	BCL2 mutations, translocations and mutations in chromatin modifiers	poor
C4	RAS/JAK/STAT and other signaling pathway abnormalities	good
C5	18q gains and MYD88 and CD79B mutation	poor

Cluster 0 lacked defining genetic drivers and included increased numbers of T-cell/histocyte-rich LBCLs.

**Table 2:** Potential therapeutic agents targeting tumor microenvironment in diffuse large B-cell lymphoma.

▪ Immune checkpoint inhibitors (e.g., anti-TIM3, anti-LAG3)
▪ anti-IL35
▪ anti-CD47 (e.g., magrolimab)
▪ Epigenetic modifiers (e.g., tazemetostat, panobinostat)

LAG3: Lymphocyte-activation-gene-3, TIM3: T cell immunoglobulin and mucin-containing molecule 3.

## Data Availability

Not applicable.

## References

[1] Sehn L.H., Salles G. (2021). Diffuse Large B-Cell Lymphoma. N. Engl. J. Med..

[2] Tilly H., Morschhauser F., Sehn L.H., Friedberg J.W., Trněný M., Sharman J.P., Herbaux C., Burke J.M., Matasar M., Rai S. (2022). Polatuzumab Vedotin in Previously Untreated Diffuse Large B-Cell Lymphoma. N. Engl. J. Med..

[3] Alizadeh A.A., Eisen M.B., Davis R.E., Ma C., Lossos I.S., Rosenwald A., Boldrick J.C., Sabet H., Tran T., Yu X. (2000). Distinct types of diffuse large B-cell lymphoma identified by gene expression profiling. Nature.

[4] Bea S., Zettl A., Wright G., Salaverria I., Jehn P., Moreno V., Burek C., Ott G., Puig X., Yang L. (2005). Diffuse large B-cell lymphoma subgroups have distinct genetic profiles that influence tumor biology and improve gene-expression-based survival prediction. Blood.

[5] Meyer P.N., Fu K., Greiner T.C., Smith L.M., Delabie J., Gascoyne R.D., Ott G., Rosenwald A., Braziel R.M., Campo E. (2011). Immunohistochemical methods for predicting cell of origin and survival in patients with diffuse large B-cell lymphoma treated with rituximab. J. Clin. Oncol..

[6] Chapuy B., Stewart C., Dunford A.J., Kim J., Kamburov A., Redd R.A., Lawrence M.S., Roemer M.G.M., Li A.J., Ziepert M. (2018). Molecular subtypes of diffuse large B cell lymphoma are associated with distinct pathogenic mechanisms and outcomes. Nat. Med..

[7] Schmitz R., Wright G.W., Huang D.W., Johnson C.A., Phelan J.D., Wang J.Q., Roulland S., Kasbekar M., Young R.M., Shaffer A.L. (2018). Genetics and Pathogenesis of Diffuse Large B-Cell Lymphoma. N. Engl. J. Med..

[8] Bai M., Skyrlas A., Agnantis N.J., Kamina S., Tsanou E., Grepi C., Galani V., Kanavaros P. (2004). Diffuse large B-cell lymphomas with germinal center B-cell-like differentiation immunophenotypic profile are associated with high apoptotic index, high expression of the proapoptotic proteins bax, bak and bid and low expression of the antiapoptotic protein bcl-xl. Mod. Pathol..

[9] Bai M., Agnantis N.J., Skyrlas A., Tsanou E., Kamina S., Galani V., Kanavaros P. (2003). Increased expression of the bcl6 and CD10 proteins is associated with increased apoptosis and proliferation in diffuse large B-cell lymphomas. Mod. Pathol..

[10] Papoudou-Bai A., Goussia A., Batistatou A., Stefanou D., Malamou-Mitsi V., Kanavaros P. (2016). The expression levels of JunB, JunD and p-c-Jun are positively correlated with tumor cell proliferation in diffuse large B-cell lymphomas. Leuk. Lymphoma.

[11] Liu Y., Zhou X., Wang X. (2021). Targeting the tumor microenvironment in B-cell lymphoma: Challenges and opportunities. J. Hematol. Oncol..

[12] Casey S.C., Amedei A., Aquilano K., Azmi A.S., Benencia F., Bhakta D., Bilsland A.E., Boosani C.S., Chen S., Ciriolo M.R. (2015). Cancer prevention and therapy through the modulation of the tumor microenvironment. Semin. Cancer Biol..

[13] Bejarano L., Jordāo M.J.C., Joyce J.A. (2021). Therapeutic Targeting of the Tumor Microenvironment. Cancer Discov..

[14] Kotlov N., Bagaev A., Revuelta M.V., Phillip J.M., Cacciapuoti M.T., Antysheva Z., Svekolkin V., Tikhonova E., Miheecheva N., Kuzkina N. (2021). Clinical and Biological Subtypes of B-cell Lymphoma Revealed by Microenvironmental Signatures. Cancer Discov..

[15] Cerchietti L. (2024). Genetic mechanisms underlying tumor microenvironment composition and function in diffuse large B-cell lymphoma. Blood.

[16] Yu T., Xu-Monette Z.Y., Lagoo A., Shuai W., Wang B., Neff J., Carrillo L.F., Carlsen E.D., Pina-Oviedo S., Young K.H. (2024). Flow cytometry quantification of tumor-infiltrating lymphocytes to predict the survival of patients with diffuse large B-cell lymphoma. Front. Immunol..

[17] Tavakkoli M., Barta S.K. (2023). 2024 Update: Advances in the risk stratification and management of large B-cell lymphoma. Am. J. Hematol..

[18] Dimou M., Papageorgiou S.G., Stavroyianni N., Katodritou E., Tsirogianni M., Kalpadakis C., Banti A., Arapaki M., Iliakis T., Bouzani M. (2021). Real-life experience with the combination of polatuzumab vedotin, rituximab, and bendamustine in aggressive B-cell lymphomas. Hematol. Oncol..

[19] Johnston R.J., Poholek A.C., DiToro D., Yusuf I., Eto D., Barnett B., Dent A.L., Craft J., Crotty S. (2009). Bcl6 and Blimp-1 are reciprocal and antagonistic regulators of T follicular helper cell differentiation. Science.

[20] Crotty S. (2014). T follicular helper cell differentiation, function, and roles in disease. Immunity.

[21] Ng W.L., Ansell S.M., Mondello P. (2022). Insights into the tumor microenvironment of B cell lymphoma. J. Exp. Clin. Cancer Res..

[22] Mintz M.A., Cyster J.G. (2020). T follicular helper cells in germinal center B cell selection and lymphomagenesis. Immunol. Rev..

[23] Cha Z., Gu H., Zang Y., Wang Z., Li J., Huang W., Qin A., Zhu L., Tu X., Cheng N. (2018). The prevalence and function of CD4^+^CXCR5^+^Foxp3^+^ follicular regulatory T cells in diffuse large B cell lymphoma. Int. Immunopharmacol..

[24] Zhou D.M., Xu Y.X., Zhang L.Y., Sun Y., Wang Z.Y., Yuan Y.Q., Fu J.X. (2017). The role of follicular T helper cells in patients with malignant lymphoid disease. Hematology.

[25] Cretney E., Kallies A., Nutt S.L. (2013). Differentiation and function of Foxp3^+^ effector regulatory T cells. Trends Immunol..

[26] Georgoulis V., Papoudou-Bai A., Makis A., Kanavaros P., Hatzimichael E. (2023). Unraveling the Immune Microenvironment in Classic Hodgkin Lymphoma: Prognostic and Therapeutic Implications. Biology.

[27] Hori S., Nomura T., Sakaguchi S. (2003). Control of regulatory T cell development by the transcription factor Foxp3. Science.

[28] Khattri R., Cox T., Yasayko S.A., Ramsdell F. (2003). An essential role for Scurfin in CD4^+^CD25^+^ T regulatory cells. Nat. Immunol..

[29] Walker M.R., Kasprowicz D.J., Gersuk V.H., Benard A., Van Landeghen M., Buckner J.H., Ziegler S.F. (2003). Induction of FoxP3 and acquisition of T regulatory activity by stimulated human CD4^+^CD25^−^ T cells. J. Clin. Investig..

[30] Yagi H., Nomura T., Nakamura K., Yamazaki S., Kitawaki T., Hori S., Maeda M., Onodera M., Uchiyama T., Fujii S. (2004). Crucial role of FOXP3 in the development and function of human CD25^+^CD4^+^ regulatory T cells. Int. Immunol..

[31] Pillai V., Ortega S.B., Wang C.K., Karandikar N.J. (2007). Transient regulatory T-cells: A state attained by all activated human T-cells. Clin. Immunol..

[32] Serag El-Dien M.M., Abdou A.G., Asaad N.Y., Abd El-Wahed M.M., Kora M. (2017). Intratumoral FOXP3^+^ Regulatory T Cells in Diffuse Large B-Cell Lymphoma. Appl. Immunohistochem. Mol. Morphol..

[33] Lee N.R., Song E.K., Jang K.Y., Choi H.N., Moon W.S., Kwon K., Lee J.H., Yim C.Y., Kwak J.Y. (2008). Prognostic impact of tumor infiltrating FOXP3 positive regulatory T cells in diffuse large B-cell lymphoma at diagnosis. Leuk. Lymphoma.

[34] Bai Y., He T., Zhang L., Liu Q., Yang J., Zhao Z., Yang K., Zhang M. (2022). Prognostic value of FOXP3^+^ regulatory T cells in patients with diffuse large B-cell lymphoma: A systematic review and meta-analysis. BMJ Open.

[35] Zhong W., Liu X., Zhu Z., Li Q., Li K. (2021). High levels of Tim-3^+^Foxp3^+^Treg cells in the tumor microenvironment is a prognostic indicator of poor survival of diffuse large B cell lymphoma patients. Int. Immunopharmacol..

[36] Zhu Q., Yang Y., Chen K., Zhang Q., Huang Y., Jian S. (2024). Diffuse large B-cell lymphoma: The significance of CD8^+^ tumor-infiltrating lymphocytes exhaustion mediated by TIM3/Galectin-9 pathway. J. Transl. Med..

[37] Collier J.L., Weiss S.A., Pauken K.E., Sen D.R., Sharpe A.H. (2021). Not-so-opposite ends of the spectrum: CD8^+^ T cell dysfunction across chronic infection, cancer and autoimmunity. Nat. Immunol..

[38] Chen Z., Deng X., Ye Y., Gao L., Zhang W., Liu W., Zhao S. (2019). Novel risk stratification of de novo diffuse large B cell lymphoma based on tumour-infiltrating T lymphocytes evaluated by flow cytometry. Ann. Hematol..

[39] Li L., Sun R., Miao Y., Tran T., Adams L., Roscoe N., Xu B., Manyam G.C., Tan X., Zhang H. (2019). PD-1/PD-L1 expression and interaction by automated quantitative immunofluorescent analysis show adverse prognostic impact in patients with diffuse large B-cell lymphoma having T-cell infiltration: A study from the International DLBCL Consortium Program. Mod. Pathol..

[40] Ansell S.M., Minnema M.C., Johnson P., Timmerman J.M., Armand P., Shipp M.A., Rodig S.J., Ligon A.H., Roemer M.G.M., Reddy N. (2019). Nivolumab for Relapsed/Refractory Diffuse Large B-Cell Lymphoma in Patients Ineligible for or Having Failed Autologous Transplantation: A Single-Arm, Phase II Study. J. Clin. Oncol..

[41] Ma J., Pang X., Li J., Zhang W., Cui W. (2022). The immune checkpoint expression in the tumor immune microenvironment of DLBCL: Clinicopathologic features and prognosis. Front. Oncol..

[42] Wang J., Sanmamed M.F., Datar I., Su T.T., Ji L., Sun J., Chen L., Chen Y., Zhu G., Yin W. (2019). Fibrinogen-like Protein 1 Is a Major Immune Inhibitory Ligand of LAG-3. Cell.

[43] Ma J., Yan S., Zhao Y., Yan H., Zhang Q., Li X. (2023). Blockade of PD-1 and LAG-3 expression on CD8+ T cells promotes the tumoricidal effects of CD8+ T cells. Front. Immunol..

[44] Wolf Y., Anderson A.C., Kuchroo V.K. (2020). TIM3 comes of age as an inhibitory receptor. Nat. Rev. Immunol..

[45] Roussel M., Le K.S., Granier C., Llamas Gutierrez F., Foucher E., Le Gallou S., Pangault C., Xerri L., Launay V., Lamy T. (2021). Functional characterization of PD1^+^TIM3^+^ tumor-infiltrating T cells in DLBCL and effects of PD1 or TIM3 blockade. Blood Adv..

[46] Xu-Monette Z.Y., Li Y., Snyder T., Yu T., Lu T., Tzankov A., Visco C., Bhagat G., Qian W., Dybkaer K. (2023). Tumor-Infiltrating Normal B Cells Revealed by Immunoglobulin Repertoire Clonotype Analysis Are Highly Prognostic and Crucial for Antitumor Immune Responses in DLBCL. Clin. Cancer Res..

[47] Yang J., Yu L., Man J., Chen H., Zhou L., Zhao L. (2023). Immune scoring model based on immune cell infiltration to predict prognosis in diffuse large B-cell lymphoma. Cancer.

[48] Vivier E., Tomasello E., Baratin M., Walzer T., Ugolini S. (2008). Functions of natural killer cells. Nat. Immunol..

[49] Hart O.M., Athie-Morales V., O’Connor G.M., Gardiner C.M. (2005). TLR7/8-mediated activation of human NK cells results in accessory cell-dependent IFN-gamma production. J. Immunol..

[50] Yokoyama W.M., Plougastel B.F. (2003). Immune functions encoded by the natural killer gene complex. Nat. Rev. Immunol..

[51] Parham P. (2005). MHC class I molecules and KIRs in human history, health and survival. Nat. Rev. Immunol..

[52] Wu S.Y., Fu T., Jiang Y.Z., Shao Z.M. (2020). Natural killer cells in cancer biology and therapy. Mol. Cancer.

[53] Cerwenka A., Lanier L.L. (2016). Natural killer cell memory in infection, inflammation and cancer. Nat. Rev. Immunol..

[54] Vari F., Arpon D., Keane C., Hertzberg M.S., Talaulikar D., Jain S., Cui Q., Han E., Tobin J., Bird R. (2018). Immune evasion via PD-1/PD-L1 on NK cells and monocyte/macrophages is more prominent in Hodgkin lymphoma than DLBCL. Blood.

[55] Wang Z., Jiang R., Li Q., Wang H., Tao Q., Zhai Z. (2021). Elevated M-MDSCs in Circulation Are Indicative of Poor Prognosis in Diffuse Large B-Cell Lymphoma Patients. J. Clin. Med..

[56] Pyzer A.R., Cole L., Rosenblatt J., Avigan D.E. (2016). Myeloid-derived suppressor cells as effectors of immune suppression in cancer. Int. J. Cancer.

[57] Sato Y., Shimizu K., Shinga J., Hidaka M., Kawano F., Kakimi K., Yamasaki S., Asakura M., Fujii S.I. (2015). Characterization of the myeloid-derived suppressor cell subset regulated by NK cells in malignant lymphoma. Oncoimmunology.

[58] Gabrilovich D.I., Ostrand-Rosenberg S., Bronte V. (2012). Coordinated regulation of myeloid cells by tumours. Nat. Rev. Immunol..

[59] Lin Y., Gustafson M.P., Bulur P.A., Gastineau D.A., Witzig T.E., Dietz A.B. (2011). Immunosuppressive CD14^+^HLA-DR(low)/^−^ monocytes in B-cell non-Hodgkin lymphoma. Blood.

[60] Nicholson L.B., Raveney B.J., Munder M. (2009). Monocyte dependent regulation of autoimmune inflammation. Curr. Mol. Med..

[61] Cripps J.G., Gorham J.D. (2011). MDSC in autoimmunity. Int. Immunopharmacol..

[62] Jaillon S., Ponzetta A., Di Mitri D., Santoni A., Bonecchi R., Mantovani A. (2020). Neutrophil diversity and plasticity in tumour progression and therapy. Nat. Rev. Cancer.

[63] Fridlender Z.G., Granot Z. (2024). Neutrophils in the tumor microenvironment—When a company becomes a crowd. Cell. Mol. Immunol..

[64] Ng M.S.F., Kwok I., Tan L., Shi C., Cerezo-Wallis D., Tan Y., Leong K., Calvo G.F., Yang K., Zhang Y. (2024). Deterministic reprogramming of neutrophils within tumors. Science.

[65] Zheng Z., Xu Y., Shi Y., Shao C. (2022). Neutrophils in the tumor microenvironment and their functional modulation by mesenchymal stromal cells. Cell. Immunol..

[66] Schwaller J., Schneider P., Mhawech-Fauceglia P., McKee T., Myit S., Matthes T., Tschopp J., Donze O., Le Gal F.A., Huard B. (2007). Neutrophil-derived APRIL concentrated in tumor lesions by proteoglycans correlates with human B-cell lymphoma aggressiveness. Blood.

[67] Manfroi B., McKee T., Mayol J.F., Tabruyn S., Moret S., Villiers C., Righini C., Dyer M., Callanan M., Schneider P. (2017). CXCL-8/IL8 Produced by Diffuse Large B-cell Lymphomas Recruits Neutrophils Expressing a Proliferation-Inducing Ligand APRIL. Cancer Res..

[68] Guidolin D., Tamma R., Annese T., Tortorella C., Ingravallo G., Gaudio F., Musto P., Specchia G., Ribatti D. (2023). Different patterns of mast cell distribution in B-cell non-Hodgkin lymphomas. Pathol. Res. Pract..

[69] Hedström G., Berglund M., Molin D., Fischer M., Nilsson G., Thunberg U., Book M., Sundström C., Rosenquist R., Roos G. (2007). Mast cell infiltration is a favourable prognostic factor in diffuse large B-cell lymphoma. Br. J. Haematol..

[70] Marinaccio C., Ingravallo G., Gaudio F., Perrone T., Nico B., Maoirano E., Specchia G., Ribatti D. (2014). Microvascular density, CD68 and tryptase expression in human diffuse large B-cell lymphoma. Leuk. Res..

[71] Patente T.A., Pinho M.P., Oliveira A.A., Evangelista G.C.M., Bergami-Santos P.C., Barbuto J.A.M. (2018). Human Dendritic Cells: Their Heterogeneity and Clinical Application Potential in Cancer Immunotherapy. Front. Immunol..

[72] Yuan C.T., Chuang S.S., Cheng P.Y., Chang K., Wang H., Tsai J.H., Liau J.Y., Chou W.C. (2022). Decreased CD11c-positive dendritic cells in the tumor microenvironment predict double-hit/triple-hit genotype and survival in diffuse large B-cell lymphoma. J. Pathol. Clin. Res..

[73] Ciavarella S., Vegliante M.C., Fabbri M., De Summa S., Melle F., Motta G., De Iuliis V., Opinto G., Enjuanes A., Rega S. (2018). Dissection of DLBCL microenvironment provides a gene expression-based predictor of survival applicable to formalin-fixed paraffin-embedded tissue. Ann. Oncol..

[74] Elhelbawy N.G., Nassar A.A.H., Eltorgoman A.E.A., Saber S.M., Badr E.A. (2020). Immunological microenvironment gene expression in patients with diffuse large B cell non Hodgkin lymphoma. Biochem. Biophys. Rep..

[75] Dallavalasa S., Beeraka N.M., Basavaraju C.G., Tulimilli S.V., Sadhu S.P., Rajesh K., Aliev G., Madhunapantula S.V. (2021). The Role of Tumor Associated Macrophages (TAMs) in Cancer Progression, Chemoresistance, Angiogenesis and Metastasis—Current Status. Curr. Med. Chem..

[76] Lin Y., Xu J., Lan H. (2019). Tumor-associated macrophages in tumor metastasis: Biological roles and clinical therapeutic applications. J. Hematol. Oncol..

[77] Sadhukhan P., Seiwert T.Y. (2023). The role of macrophages in the tumor microenvironment and tumor metabolism. Semin. Immunopathol..

[78] Cioroianu A.I., Stinga P.I., Sticlaru L., Cioplea M.D., Nichita L., Popp C., Staniceanu F. (2019). Tumor Microenvironment in Diffuse Large B-Cell Lymphoma: Role and Prognosis. Anal. Cell. Pathol..

[79] Khalifa K.A., Badawy H.M., Radwan W.M., Shehata M.A., Bassuoni M.A. (2014). CD14^+^ HLA-DR low/^−^ monocytes as indicator of disease aggressiveness in B-cell non-Hodgkin lymphoma. Int. J. Lab. Hematol..

[80] Marchesi F., Cirillo M., Bianchi A., Gately M., Olimpieri O.M., Cerchiara E., Renzi D., Micera A., Balzamino B.O., Bonini S. (2015). High density of CD68^+^/CD163^+^ tumour-associated macrophages (M2-TAM) at diagnosis is significantly correlated to unfavorable prognostic factors and to poor clinical outcomes in patients with diffuse large B-cell lymphoma. Hematol. Oncol..

[81] Nam S.J., Go H., Paik J.H., Kim T.M., Heo D.S., Kim C.W., Jeon Y.K. (2014). An increase of M2 macrophages predicts poor prognosis in patients with diffuse large B-cell lymphoma treated with rituximab, cyclophosphamide, doxorubicin, vincristine and prednisone. Leuk. Lymphoma.

[82] Wada N., Zaki M.A., Hori Y., Hashimoto K., Tsukaguchi M., Tatsumi Y., Ishikawa J., Tominaga N., Sakoda H., Take H. (2012). Tumour-associated macrophages in diffuse large B-cell lymphoma: A study of the Osaka Lymphoma Study Group. Histopathology.

[83] Hasselblom S., Hansson U., Sigurdardottir M., Nilsson-Ehle H., Ridell B., Andersson P.O. (2008). Expression of CD68^+^ tumor-associated macrophages in patients with diffuse large B-cell lymphoma and its relation to prognosis. Pathol. Int..

[84] Meyer P.N., Fu K., Greiner T., Smith L., Delabie J., Gascoyne R., Ott G., Rosenwald A., Braziel R., Campo E. (2011). The stromal cell marker SPARC predicts for survival in patients with diffuse large B-cell lymphoma treated with rituximab. Am. J. Clin. Pathol..

[85] Cai Q.C., Liao H., Lin S.X., Xia Y., Wang X.X., Gao Y., Lin Z.X., Lu J.B., Huang H.Q. (2012). High expression of tumor-infiltrating macrophages correlates with poor prognosis in patients with diffuse large B-cell lymphoma. Med. Oncol..

[86] Riihijärvi S., Fiskvik I., Taskinen M., Vajavaara H., Tikkala M., Yri O., Karjalainen-Lindsberg M.L., Delabie J., Smeland E., Holte H. (2015). Prognostic influence of macrophages in patients with diffuse large B-cell lymphoma: A correlative study from a Nordic phase II trial. Haematologica.

[87] Sun X., Cao J., Sun P., Yang H., Li H., Ma W., Wu X., He X., Li J., Li Z. (2023). Pretreatment soluble Siglec-5 protein predicts early progression and R-CHOP efficacy in diffuse large B-cell lymphoma. Biomark. Med..

[88] Nikkarinen A., Lokhande L., Amini R.M., Jerkeman M., Porwit A., Molin D., Enblad G., Kolstad A., Räty R., Hutchings M. (2023). Soluble CD163 predicts outcome in both chemoimmunotherapy and targeted therapy-treated mantle cell lymphoma. Blood Adv..

[89] Koudouna A., Gkioka A.I., Gkiokas A., Tryfou T.M., Papadatou M., Alexandropoulos A., Bartzi V., Kafasi N., Kyrtsonis M.C. (2024). Serum-Soluble CD163 Levels as a Prognostic Biomarker in Patients with Diffuse Large B-Cell Lymphoma Treated with Chemoimmunotherapy. Int. J. Mol. Sci..

[90] Chao M.P., Alizadeh A.A., Tang C., Myklebust J.H., Varghese B., Gill S., Jan M., Cha A.C., Chan C.K., Tan B.T. (2010). Anti-CD47 antibody synergizes with rituximab to promote phagocytosis and eradicate non-Hodgkin lymphoma. Cell.

[91] Eladl E., Tremblay-LeMay R., Rastgoo N., Musani R., Chen W., Liu A., Chang H. (2020). Role of CD47 in Hematological Malignancies. J. Hematol. Oncol..

[92] Kazama R., Miyoshi H., Takeuchi M., Miyawaki K., Nakashima K., Yoshida N., Kawamoto K., Yanagida E., Yamada K., Umeno T. (2020). Combination of CD47 and signal-regulatory protein-α constituting the “don’t eat me signal” is a prognostic factor in diffuse large B-cell lymphoma. Cancer Sci..

[93] Maakaron J., Asch A.S., Popplewell L.L., Collins G.P., Flinn I.W., Ghosh N., Keane C., Ku M., Mehta A., Roschewski M. (2022). Magrolimab in Combination with Rituximab + Chemotherapy in Patients with Relapsed or Refractory (R/R) Diffuse Large B-Cell Lymphoma (DLBCL). Blood.

[94] Kalluri R. (2016). The biology and function of fibroblasts in cancer. Nat. Rev. Cancer.

[95] Yang D., Liu J., Qian H., Zhuang Q. (2023). Cancer-associated fibroblasts: From basic science to anticancer therapy. Exp. Mol. Med..

[96] Öhlund D., Handly-Santana A., Biffi G., Elyada E., Almeida A.S., Ponz-Sarvise M., Corbo V., Oni T.E., Hearn S.A., Lee E.J. (2017). Distinct populations of inflammatory fibroblasts and myofibroblasts in pancreatic cancer. J. Exp. Med..

[97] Elyada E., Bolisetty M., Laise P., Flynn W.F., Courtois E.T., Burkhart R.A., Teinor J.A., Belleau P., Biffi G., Lucito M.S. (2019). Cross-Species Single-Cell Analysis of Pancreatic Ductal Adenocarcinoma Reveals Antigen-Presenting Cancer-Associated Fibroblasts. Cancer Discov..

[98] Haro M., Orsulic S. (2018). A Paradoxical Correlation of Cancer-Associated Fibroblasts With Survival Outcomes in B-Cell Lymphomas and Carcinomas. Front. Cell Dev. Biol..

[99] Lenz G., Wright G., Dave S.S., Xiao W., Powell J., Zhao H., Xu W., Tan B., Goldschmidt N., Iqbal J. (2008). Stromal gene signatures in large-B-cell lymphomas. N. Engl. J. Med..

[100] Frazier K., Williams S., Kothapalli D., Klapper H., Grotendorst G.R. (1996). Stimulation of fibroblast cell growth, matrix production, and granulation tissue formation by connective tissue growth factor. J. Investig. Dermatol..

[101] Sangaletti S., Tripodo C., Portararo P., Dugo M., Vitali C., Botti L., Guarnotta C., Cappetti B., Gulino A., Torselli I. (2014). Stromal niche communalities underscore the contribution of the matricellular protein SPARC to B-cell development and lymphoid malignancies. Oncoimmunology.

[102] Chlenski A., Cohn S.L. (2010). Modulation of matrix remodeling by SPARC in neoplastic progression. Semin. Cell Dev. Biol..

[103] Brandt S., Montagna C., Georgis A., Schüffler P.J., Bühler M.M., Seifert B., Thiesler T., Curioni-Fontecedro A., Hegyi I., Dehler S. (2013). The combined expression of the stromal markers fibronectin and SPARC improves the prediction of survival in diffuse large B-cell lymphoma. Exp. Hematol. Oncol..

[104] Takahara T., Nakamura S., Tsuzuki T., Satou A. (2023). The Immunology of DLBCL. Cancers.

[105] Raskov H., Orhan A., Christensen J.P., Gögenur I. (2021). Cytotoxic CD8^+^ T cells in cancer and cancer immunotherapy. Br. J. Cancer.

[106] Fangazio M., Ladewig E., Gomez K., Garcia-Ibanez L., Kumar R., Teruya-Feldstein J., Rossi D., Filip I., Pan-Hammarström Q., Inghirami G. (2021). Genetic mechanisms of HLA-I loss and immune escape in diffuse large B cell lymphoma. Proc. Natl. Acad. Sci. USA.

[107] Challa-Malladi M., Lieu Y.K., Califano O., Holmes A.B., Bhagat G., Murty V.V., Dominguez-Sola D., Pasqualucci L., Dalla-Favera R. (2011). Combined genetic inactivation of β2-Microglobulin and CD58 reveals frequent escape from immune recognition in diffuse large B cell lymphoma. Cancer Cell.

[108] Axelrod M.L., Cook R.S., Johnson D.B., Balko J.M. (2019). Biological Consequences of MHC-II Expression by Tumor Cells in Cancer. Clin. Cancer Res..

[109] Wilkinson S.T., Vanpatten K.A., Fernandez D.R., Brunhoeber P., Garsha K.E., Glinsmann-Gibson B.J., Grogan T.M., Teruya-Feldstein J., Rimsza L.M. (2012). Partial plasma cell differentiation as a mechanism of lost major histocompatibility complex class II expression in diffuse large B-cell lymphoma. Blood.

[110] Bertrand P., Maingonnat C., Penther D., Guney S., Ruminy P., Picquenot J.M., Mareschal S., Alcantara M., Bouzelfen A., Dubois S. (2013). The costimulatory molecule CD70 is regulated by distinct molecular mechanisms and is associated with overall survival in diffuse large B-cell lymphoma. Genes Chromosomes Cancer.

[111] Denoeud J., Moser M. (2011). Role of CD27/CD70 pathway of activation in immunity and tolerance. J. Leukoc. Biol..

[112] Honikel M.M., Olejniczak S.H. (2022). Co-Stimulatory Receptor Signaling in CAR-T Cells. Biomolecules.

[113] Modi D., Potugari B., Uberti J. (2021). Immunotherapy for Diffuse Large B-Cell Lymphoma: Current Landscape and Future Directions. Cancers.

[114] Ennishi D., Takata K., Béguelin W., Duns G., Mottok A., Farinha P., Bashashati A., Saberi S., Boyle M., Meissner B. (2019). Molecular and Genetic Characterization of MHC Deficiency Identifies EZH2 as Therapeutic Target for Enhancing Immune Recognition. Cancer Discov..

[115] Munakata W., Shirasugi Y., Tobinai K., Onizuka M., Makita S., Suzuki R., Maruyama D., Kawai H., Izutsu K., Nakanishi T. (2021). Phase 1 study of tazemetostat in Japanese patients with relapsed or refractory B-cell lymphoma. Cancer Sci..

[116] Palomba M.L., Cartron G., Popplewell L., Ribrag V., Westin J., Huw L.Y., Agarwal S., Shivhare M., Hong W.J., Raval A. (2022). Combination of Atezolizumab and Tazemetostat in Patients with Relapsed/Refractory Diffuse Large B-Cell Lymphoma: Results From a Phase Ib Study. Clin. Lymphoma Myeloma Leuk..

[117] Zhang J., Gu Y., Chen B. (2023). Drug-Resistance Mechanism and New Targeted Drugs and Treatments of Relapse and Refractory DLBCL. Cancer Manag. Res..

[118] Wang X., Waschke B.C., Woolaver R.A., Chen S.M.Y., Chen Z., Wang J.H. (2020). HDAC inhibitors overcome immunotherapy resistance in B-cell lymphoma. Protein Cell.

[119] Assouline S.E., Nielsen T.H., Yu S., Alcaide M., Chong L., MacDonald D., Tosikyan A., Kukreti V., Kezouh A., Petrogiannis-Haliotis T. (2016). Phase 2 study of panobinostat with or without rituximab in relapsed diffuse large B-cell lymphoma. Blood.

[120] Islam P., Rizzieri D., Lin C., de Castro C., Diehl L., Li Z., Moore J., Morris T., Beaven A. (2021). Phase II Study of Single-Agent and Combination Everolimus and Panobinostat in Relapsed or Refractory Diffuse Large B-Cell Lymphoma. Cancer Investig..

[121] Godfrey J., Mei M., Chen L., Song J.Y., Bedell V., Budde E., Armenian S., Puverel S., Nikolaenko L., Chen R. (2024). Results from a phase I trial of pembrolizumab plus vorinostat in relapsed/refractory B-cell non-Hodgkin lymphoma. Haematologica.

[122] Opinto G., Vegliante M.C., Negri A., Skrypets T., Loseto G., Pileri S.A., Guarini A., Ciavarella S. (2020). The Tumor Microenvironment of DLBCL in the Computational Era. Front. Oncol..

[123] Mackay F., Schneider P. (2009). Cracking the BAFF code. Nat. Rev. Immunol..

[124] Shain K.H., Dalton W.S., Tao J. (2015). The tumor microenvironment shapes hallmarks of mature B-cell malignancies. Oncogene.

